# A Different Method to Increase Breast Projection—Tunnelized Glandular Flap

**DOI:** 10.1007/s00266-024-03986-3

**Published:** 2024-03-28

**Authors:** Can Kopal, Ilker Uyar, Ersin Aksam

**Affiliations:** 1 Private Practice, Kultur Mah, Sair Esref Bul, No:61 Bahar Apartmanı K:2 D:5, Alsancak/İzmir, Turkey; 2 Department of Plastic, Reconstructive and Aesthetic Surgery, Izmir Katip Celebi University Medical Faculty, Izmir Katip Celebi University, Basın Sitesi, Karabağlar, 35360 İzmir, Turkey

**Keywords:** Breast lift, Glandular flap, Projection

## Abstract

**Background:**

Breast ptosis may occur with increasing age, after pregnancy, after breastfeeding, or after weight loss. Understanding the vascular structure of the breast and nipple-areolar complex has guided the reshaping of the breast and thus paved the way for the emergence of different techniques. This study aimed to evaluate the results of tunneled glandular flaps used to increase projection in patients undergoing breast lift surgery.

**Methods:**

Patients who underwent breast lift and breast reduction between January 2020 and January 2022 were examined through their files and included in the study. Deepithelialization of the superomedial pedicle was performed. A tunnel was created under the pedicle. A medial or lateral based glandular flap was prepared from the inferir. The prepared glandular flap was passed through the tunnel and fixed to the pectoral muscle.

**Results:**

A total of 32 patients were included in the study. The average age of the patients was 44.31. Thirteen patients were smokers. Diabetes mellitus was present in 5 patients. To increase projection, medial glandular flap was used in 20 patients and lateral glandular flap was used in 12 patients. The average amount of tissue excised from the patients was 785.31 g. The average follow-up period was 14 months.

**Conclusions:**

Tunneled glandular flaps prepared on a lateral or medial basis will be useful in increasing the projection in breast lift surgery.

**Level of Evidence V:**

This journal requires that authors assign a level of evidence to each article. For a full description of these Evidence-Based Medicine ratings, please refer to the Table of Contents or the online Instructions to Authors www.springer.com/00266.

**Supplementary Information:**

The online version contains supplementary material available at 10.1007/s00266-024-03986-3.

## Introduction

Breast ptosis may occur with increasing age, after pregnancy, after breastfeeding, or after weight loss. The Regnault classification is frequently used to grade ptosis. In this classification, nipple-areolar complex and inframalar fold positions are evaluated [1–5].

Understanding the vascular structure of the breast and nipple-areolar complex has guided the reshaping of the breast and thus paved the way for the emergence of different techniques [6–11].

The vertical scar breast reduction technique was popularized by Lejour, who used the superior pedicle in this technique [12]. In the following years, different pedicles and modifications of these pedicles took their place in books [13]. The goals expected to be achieved at the end of the operation in all these techniques are as follows: safe neurovascular supply in the nipple-areolar complex, quality lactation, good upper pole fullness, less scarring on the skin and shortening the healing time [13–15].

In the technique using the central pedicle, it is aimed to feed the nipple-areolar complex from the base, while in other techniques, the supply is from the glandular part carrying the pedicle [16].

Projection of the breast is one of the primary targets in the breast lift surgeries. Although different methods for projection have been described, the intraoperative and long-term results of these methods are not fully satisfactory [13].

This study aimed to evaluate the results of tunneled glandular flaps used to increase projection in patients undergoing breast lift surgery.

## Material and Methods

The study was planned retrospectively. Ethics committee approval was obtained for this study by xxx University Ethics Committee, and it was prepared following the Declaration of Helsinki. Informed consent forms were obtained before surgery from the patients or their legal representatives if necessary. Patients who underwent breast lift and breast reduction surgeries between January 2020 and January 2022 were examined through their files and included in the study. Demographic information, comorbidities and smoking status of the patients were examined through patient files. Patients who did not comply with postoperative recommendations and attend regular check-ups were excluded from the study. The same surgeon performed all operations and data collection (CK). All surgeries were performed under general anesthesia. Superomedial pedicle wise-pattern breast reduction technique was applied to all patients. In preoperative evaluations, the breast tissue to be excised was determined, and after the pedicle skeletonization, the excess glandular tissue was prepared as a inferomedial or inferolateral based flap. In all patients, after excision, the repair was performed in a double layer, and a strip (Hartman, Germany) was adhered to the suture line. In the postoperative period, empirical antibiotic therapy was administered to the patients. Patients were followed regularly for at least 12 months. To evaluate the results, patients were called for regular check-ups, a physical examination and ultrasonography were performed at the 6th month, and the data were recorded.

### Surgical Technique

**Fig 1:**
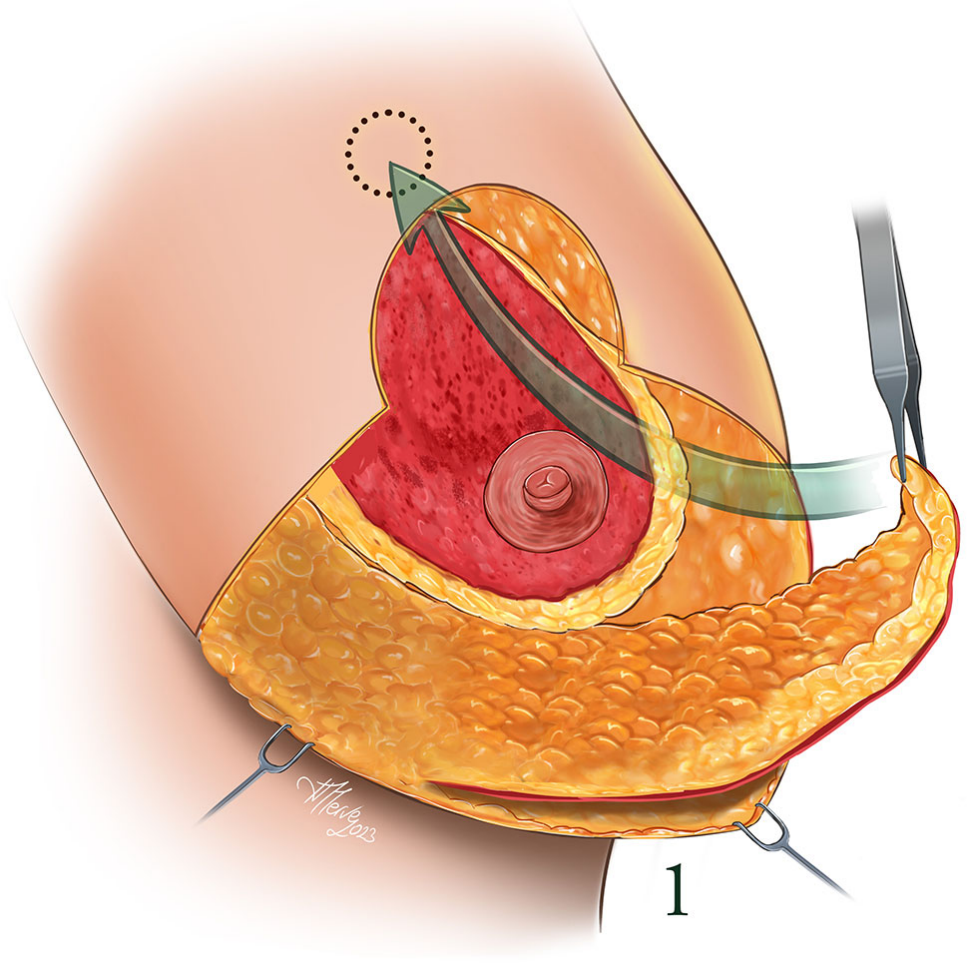
Prepared glandular flap

**Fig 2:**
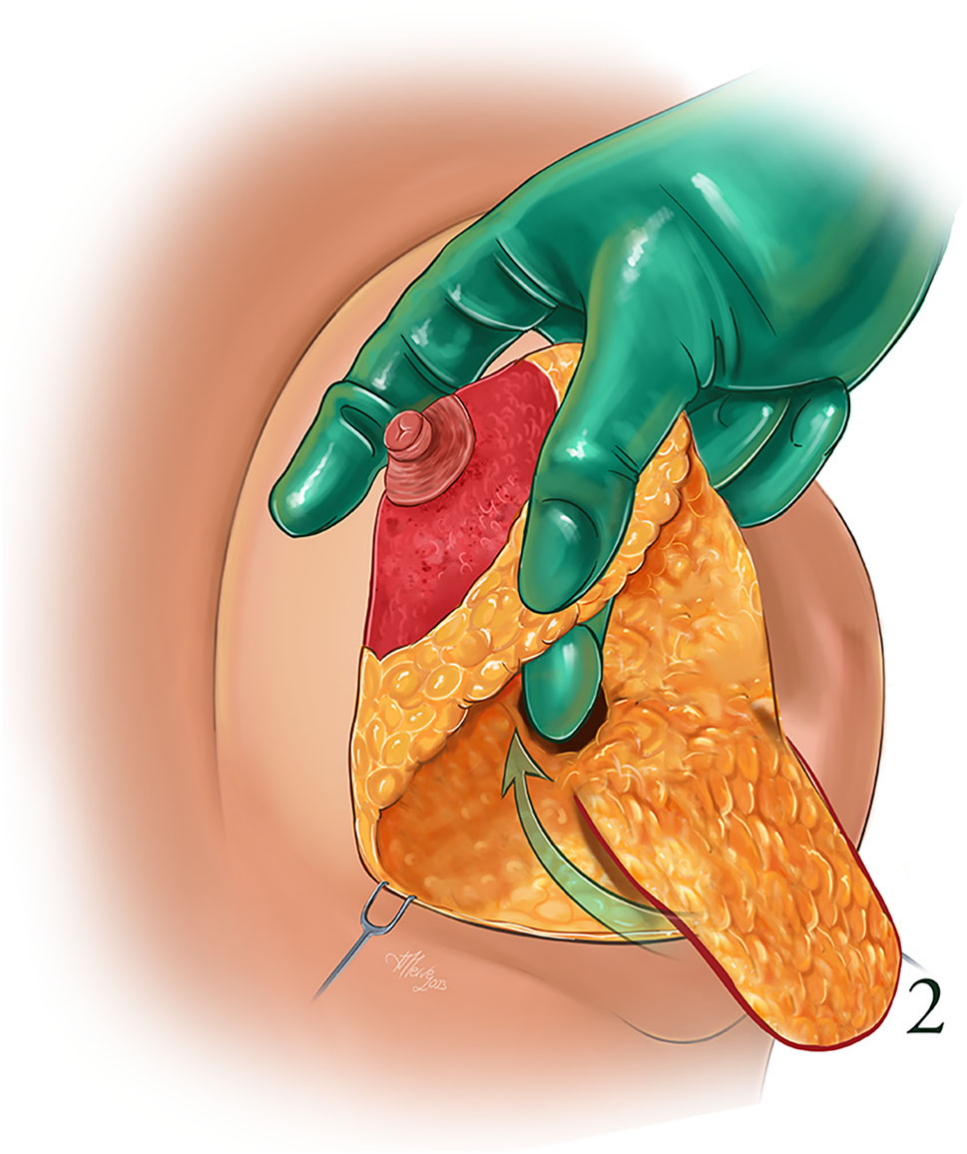
Tunnel created under the pedicle for the glandular flap

An anesthetic solution (Jetokaine) containing lidocaine and adrenaline was infiltrated into the incision lines in accordance with the preoperative drawings. Deepithelialization of the superomedial pedicle was performed. Then, a skin incision was made in accordance with the vertical mammoplasty technique and the pedicle was skeletonized. A tunnel was created under the superomedial pedicle. A medial or lateral based glandular flap was prepared from the inferolateral breast tissue to be excised, and the area outside the glandular flap was excised. The flap prepared from the inferolateral or inferomedial is 14–18 cm in lenght and 6–8 cm in width. Since flap circulation is extremely strong, it is very safe. The prepared glandular flap was passed through the tunnel created under the pedicle and fixed to the pectoral muscle with 2/0 polydioxanone (PDS, Ethicon) (Figs. 1, 2). After bleeding control, the nipples were positioned in their new places, the skin flaps were fixed in their new places so that the nipple-inframamarian fold distance was 8–10 cm compared to the patient’s rib cage, subcutaneous sutures were performed with 2/0 polyglytone (Caprosyn, Covidien), skin sutures were performed, with 3/0 polyglytone (Caprosyn, Covidien). The excess skin in the inframaternal fold was sutured in the form of a purse string. Surgical drains were not used in all patients. After sutures, strips (Hartman, Germany) were applied to the operation area. Antibiotics were administered to the patients in the postoperative period. The strips were removed on the 20th postoperative day, and scar treatment (Dermatix Si Gel) was started for all patients. (Video 1)

### Statistical Analyses

IBM SPSS Statistics version 20 (IBM, USA) was used for statistical analysis. The Shapiro-Wilk test was used for normality analysis. Chi-square test was used for binomial values, independent samples *T*-test and Mann-Whitney *U* test were used for other values. Statistical significance was set as *p* < 0.05.

## Results

A total of 70 patients were operated on for breast reduction or lift between January 2020 and January 2022. Thirty-eight of these patients were excluded from the study because they did not comply with postoperative recommendations and did not attend regular check-ups. A total of 32 patients were included in the study. Breast lift was performed in 11 patients, and breast reduction was performed in 21 patients. The average age of the patients was 44.31. Thirteen patients were smokers. Diabetes mellitus was present in five patients. To increase projection, medial glandular flap was used in 20 patients and lateral glandular flap was used in 12 patients. The average amount of tissue excised from the patients was 785.31 g. The average follow-up period was 14 months. No problems such as bleeding, hematoma, dehiscence, seroma, or fat necrosis were detected in any patient in the postoperative period. Delayed wound healing was detected in the purse-string suture area in 14 patients, and full recovery was achieved with appropriate wound care in these patients. Ten patients underwent scar revision for the area where the purse string was applied at the 6th postoperative month (Table 1).

**Table 1:** Demographics of the patients

Patient number	Age	Smoking	Komorbidity	Pedicle	Weight of excised tissue	Follow up (month)	Complication	Revision surgery
1	32	−	None	Medial	450	13	None	−
2	43	+	None	Medial	400	12	None	−
3	37	+	None	Medial	720	12	None	−
4	45	−	None	Medial	560	14	None	−
5	41	+	None	Medial	830	12	Delay in wound healing	−
6	38	−	None	Lateral	680	15	None	−
7	40	+	None	Medial	530	12	None	−
8	47	+	DM	Lateral	900	13	Delay in wound healing	+
9	39	−	None	Lateral	1040	16	Delay in wound healing	+
10	45	−	None	Medial	850	14	None	+
11	44	+	None	Medial	470	12	None	−
12	52	−	None	Lateral	1200	18	Delay in wound healing	+
13	48	−	DM	Lateral	1000	13	Delay in wound healing	+
14	35	+	None	Medial	950	14	Delay in wound healing	−
15	46	−	None	Lateral	600	15	None	−
16	49	+	None	Lateral	630	14	None	−
17	50	−	DM	Lateral	1100	16	Delay in wound healing	+
18	51	+	None	Medial	770	12	None	−
19	43	−	DM	Medial	840	12	Delay in wound healing	−
20	38	+	None	Medial	800	14	None	−
21	50	+	None	Medial	1000	19	Delay in wound healing	+
22	53	−	None	Medial	1200	15	Delay in wound healing	+
23	52	−	None	Lateral	950	14	Delay in wound healing	−
24	45	+	None	Lateral	820	16	Delay in wound healing	−
25	47	−	None	Medial	930	12	None	−
26	42	−	DM	Medial	860	17	Delay in wound healing	−
27	45	−	None	Medial	620	13	None	+
28	35	−	None	Lateral	750	14	None	−
29	45	+	None	Medial	480	13	None	−
30	43	−	None	Medial	910	16	Delay in wound healing	+
31	48	−	None	Medial	530	14	None	−
32	50	−	None	Lateral	760	12	None	−

In the statistical analysis, no significant difference was found between smoking and complication development. No significant difference was found between smoking and revision surgery. No significant difference was found between comorbidity and revision surgery.

**Fig 3:**
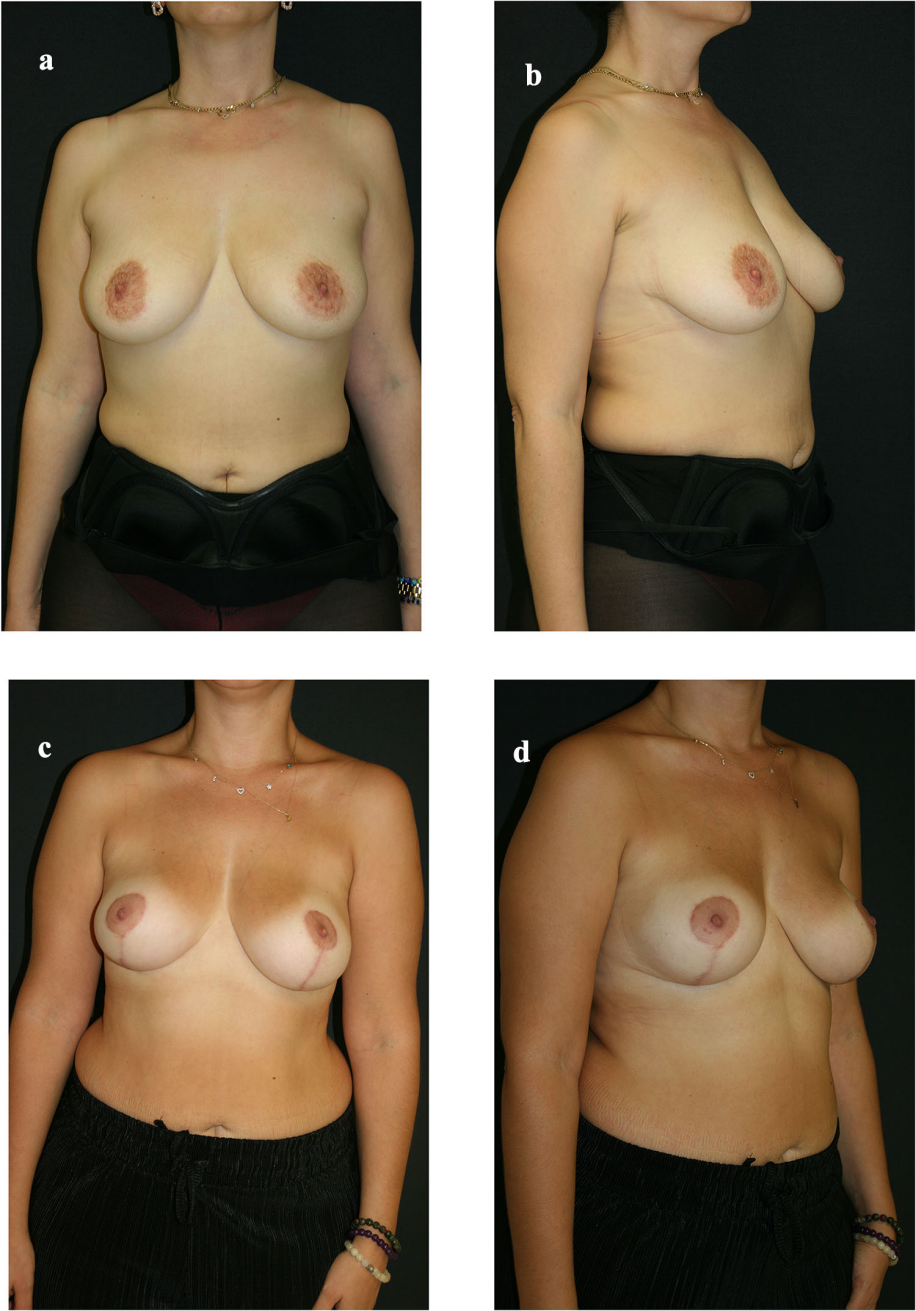
Preoperative and postoperative views of the patient #2. **a** Preoperative front view of Patient #2. **b** Preoperative lateral view of Patient #2. **c** Postoperative front view of Patient #2. **d** Postoperative lateral view of Patient #2

**Fig 4:**
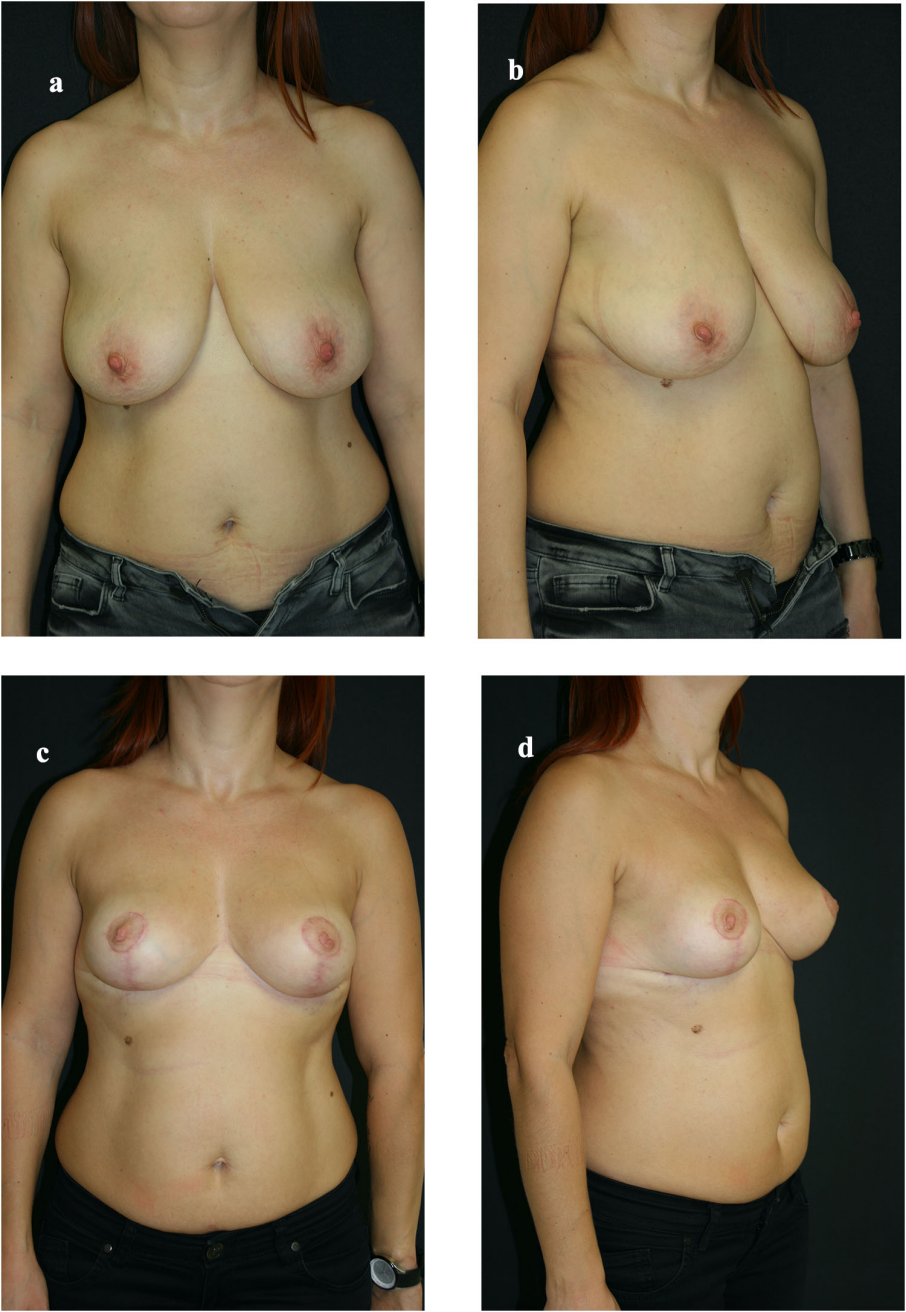
Preoperative and postoperative views of the patient #7. **a** Preoperative front view of Patient #7. **b** Preoperative lateral view of Patient #7. **c** Postoperative front view of Patient #7. **d** Postoperative lateral view of Patient #7

**Fig 5:**
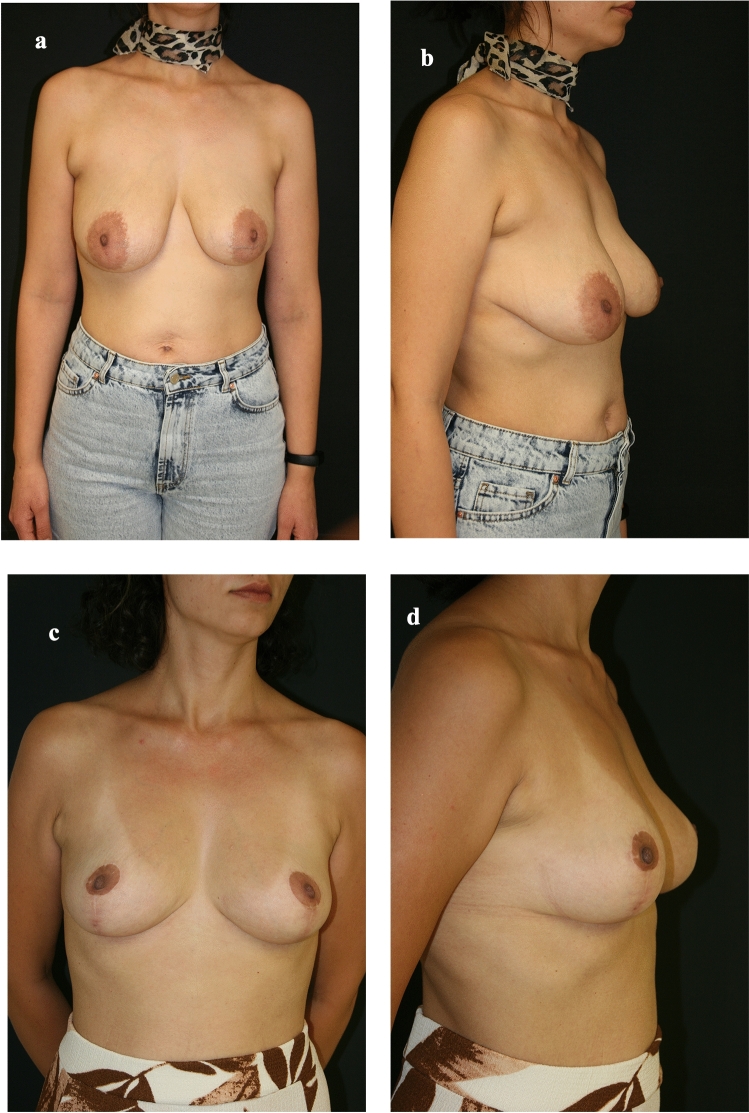
Preoperative and postoperative views of the patient #29. **a** Preoperative front view of Patient #29. **b** Preoperative lateral view of Patient #29. **c** Postoperative front view of Patient #29. **d** Postoperative lateral view of Patient #29

A statistically significant difference was found between comorbidity and complications (*p* = 0.01) (Table 2) (Figs. 3, 4 and 5).

**Table 2:** Statistical analyzes

	Complication	Revision surgery
Smoking (*n*=13) %40.6	0.725	0.141
Comorbidity (*n*=5) %15.6	0.010	0.293

## Discussion

Breast is a symbol of femininity and motherhood for women. Breast deformities caused by reasons such as aging, postnatal growth and involution, and breastfeeding are among the conditions that women frequently complain about, and this causes both functional and esthetic concerns [1–5].

Macromastia and breast ptosis are common conditions that negatively affect the quality of life in women, and breast reduction-lift surgeries are among the most frequently performed esthetic surgeries all over the world [18].

The strategy in breast reduction-lift surgeries is based on shaping the breast gland and minimal scarring. The goals of these techniques are to ensure upper pole fullness, good projection, safety of the nipple-areolar complex and lactation, not to see bottoming-out deformity in the early period, and to have minimal scarring [19]. In many techniques, fullness does not occur in the upper pole and recurrent ptosis is observed [20–22].

In wise-pattern breast reduction, the shape is close to straight. In the vertical technique, the projection may be better, but the NAC-IMF distance may be longer [23]. Different autoaugmentation techniques have been described in the literature, and in these techniques, the gland tissue is separated from the pectoral muscle and tunneled, leading to different complications, especially fat necrosis and hematoma [24].

There are different breast lift techniques in the literature using glandular breast tissue [25–27]. In a study by Yılmaz, a triangular-shaped dermoglandular flap was prepared from the inferolateral side and transposed superiorly. In this study, it was applied only in cases where the superior pedicle was used, and the size of the prepared flap was small [27]. In the technique we described, flaps can be prepared both inferolaterally and inferomedially, and the flaps are larger in size. Therefore, superior hanging is more comfortable in extremely sagging breasts.

In a study conducted by Kotti, the breast tissue inferior to the NAC was divided into two, the lateral tissue was sutured under the NAC, and the medial tissue was combined with the lateral [28]. The lateral-based flap prepared in this study does not reach the upper pole of the breast, it only extends below the NAC, and only the superior pedicle was used [28]. In our study, the prepared flaps also provide fullness in the upper pole.

In a study conducted by Sozer and Philips in 2021, a myoglandular flap was created based on the glandular parenchyma and pectoralis major muscle, and this flap was advanced under the pectoral muscle and hung on the perichondrium [29]. In this study, it is claimed that the neurovascular safety of the NAC is preserved and the risk of necrosis is very low because the myoglandular flap is unblocked [29]. When the myoglandular flap is under the pectoralis muscle, the pressure on the myoglandular flap will increase during movements of the pectoralis muscle, causing thinning of the pedicle and thus decreasing the projection over time. In addition, in this technique, there is a risk of increase in fat necrosis secondary to pressure under the pectoralis muscle in breasts with high fat content.The glandular flaps we created in our study were advanced directly over the pectoral muscle, not under the pectoral muscle, and were fixed superiorly on the muscle. In this way, muscle compression is prevented. No neurovascular problems were detected in NAC in any patient. It was observed intraoperatively that the glandular flaps separated and advanced over the pectoral muscle were also viable. In this study, tunneled glandular flaps were used to increase projection in patients who underwent vertical scar breast reduction with superomedial pedicle. Flaps were lifted laterally or medially from the inferior area outside the pedicle and passed through the tunnel created at the base of the pedicle and hung superiorly to the thorax wall. In this way, breast projection is increased with the help of tissues normally excised from the breast without the use of any implants. In our study, there were no statistically significant results in terms of smoking-complication, smoking-revision surgery, comorbidity-revision surgery, but a statistically significant difference was found between comorbidity-revision surgery. In other words, the possibility of dehiscence is higher in those with comorbidities, as expected, but this can be overcome with appropriate wound care and resuturation.

The advantage of this study is that it does not require any implant to provide projection and the results can be seen immediately intraoperatively. The disadvantage of the study is the limited number of cases. Different statistical results may occur in higher number of patients. Another disadvantage of the study is that it may cause a secondary scar in patients in whom only the vertical scar technique can be used. However, if desired, this operation can be performed safely using the wise-pattern technique. Additionally, longer-term follow-ups may be done for patients.

## Conclusion

As a result, we believe that tunneled glandular flaps prepared on a lateral or medial basis will be useful in increasing the projection in breast lift surgery.

## Supplementary Information

Below is the link to the electronic supplementary material.

Video 1: The glandular flap is prepared inferiorly on a lateral or medial basis. Then, after passing through the tunnel created under the pedicle, it is suspended superiorly to increase breast projection. (MP4 2922 KB)
